# Recent advances in understanding and managing pituitary adenomas

**DOI:** 10.12703/r/12-6

**Published:** 2023-03-21

**Authors:** Maria Markou, Aikaterini Lavrentaki, Georgia Ntali

**Affiliations:** 1 Private Endocrinology, Diabetes and Metabolism Clinic, Daidalou 11, Levadia, 32131, Greece; 2 Department of Endocrinology, Diabetes and Metabolism, Evangelismos Hospital, 10676 Athens, Greece

**Keywords:** Pituitary adenomas, tumorigenesis, transsphenoidal neurosurgery, medical treatment, radiotherapy, targeted systemic treatment

## Abstract

Pituitary adenomas (PAs) are common intracranial tumors. Despite their benign nature, PAs may cause a significant burden of disease, leading to either hormonal disturbances or local compression. A subset of PAs presents an aggressive behavior that remains difficult to predict, and in rare cases they metastasize. Therefore, early diagnosis and treatment are important. Advances in molecular pathology have improved the understanding of their pathogenesis and offer opportunities to identify and target novel pathways. Improved imaging and functional molecular techniques precisely detect even very small tumors and guide targeted treatment. Transsphenoidal surgery is the first-line treatment for the majority of PAs, and advances in the field of endoscopic neurosurgery offer excellent outcomes. Dopamine agonists (DAs) are traditionally the first-line treatment for prolactinomas. For patients with acromegaly, first- and second-generation somatostatin analogues (SSAs) are applied when surgery is not successful or not indicated. For Cushing’s disease (CD), drugs targeting adrenal steroidogenesis, somatostatin receptors in the pituitary, and glucocorticoid receptors are used to treat hypercortisolism in patients with persistent or recurrent CD, for those who are not good surgical candidates, and as a bridge treatment for those who have undergone radiation treatment until cortisol levels are controlled. Temozolomide (TMZ) is the first-line chemotherapy for aggressive PAs, but new experimental therapies, like the anti-vascular endothelial growth factor (anti-VEGF) therapy, mechanistic target of rapamycin (mTOR) inhibitors, tyrosine kinase inhibitors, and cell cycle and checkpoint inhibitors, are now available. Radiotherapy is offered to patients with residual, recurrent, or progressive tumors. Modern techniques in radiotherapy planning and delivery are able to deliver high doses to the target tissue while sparing vital structures. As we familiarize ourselves with the biological behavior of PAs and our therapeutic armamentarium expands, the next goal is to tailor and personalize treatment to each individual patient so as to achieve the best outcome.

## Introduction

Pituitary adenomas (PAs) are the second most common brain tumors^1^, classified according to their functionality, size, and anatomical extension. In the general population, epidemiologic data show an increasing incidence (3.9–7.4 cases per 100,000/year) and prevalence (76–116 cases per 100,000 population), likely related to increasing magnetic resonance imaging (MRI) usage^2^. Despite their benign nature, PAs can occasionally cause significant morbidity and mortality, and 0.2% of PAs can present metastasis. According to European Society of Endocrinology (ESE) guidelines from 2018, aggressive pituitary tumors are large and invasive tumors that grow rapidly despite conventional therapies, such as surgery, radiotherapy, or standard medical treatment, including TMZ^3^. Clinical presentation of PAs is related to hormone excess, hypopituitarism, or pressure effects. First-line therapy for the majority of PAs is transsphenoidal surgery. Pharmacotherapy and radiotherapy have an additional role in cases of hormone secreting or invasive, unresectable adenomas, or adenomas that are too small to be detected. However, as recurrences are not uncommon even in cases of initial successful pituitary surgery, it is essential to comprehend in depth the genetic background of these tumors and identify potential “drivers of tumorigenesis”, to familiarize oneself with their biological characteristics in order to make an early diagnosis, an accurate prognosis, and a targeted and effective treatment plan. In this review, we describe recent advances in understanding the pathogenesis of PAs and novel insights into their diagnostic and therapeutic management. (The most important are provided in Table 1.)

**Table 1.** An overview of recent advances in pathophysiology and management of pituitary adenomas.

	Pathophysiology	Novel treatment (*in vivo* or *in vitro*)
**Non-functioning pituitary** ** adenomas (NFPAs), null cell** ** adenomas** **and gonadotroph adenomas**	Non-coding RNAs (LOC101927765, RP11-23N2.4, and RP4-533D7.4) have been associated with NFPA recurrence Upregulation of miR582, miR4774, and LINC01351	Cabergoline Cabergoline + everolimus Chimeric compound TBR-760
**Prolactinοmas**	Oncogenic mutation SF3B1R625H Overexpression of miR377 and miR136 Wnt/β-catenin pathway mutation Expression of specific ErbB receptors PI3K/Akt/mechanistic target of rapamycin (mTOR) pathway	Bromocriptine + fulvestrant Octreotide long-acting repeatable (LAR) + cabergoline Pasireotide Metformin Anastrozole Temozolomide + capecitabine Lapatinib Ipilimumab + nivolumab Everolimus ± cabergoline Surgery G-knife radiosurgery
**Somatotroph adenomas**	Germline mutations AIP, PRKAR1A, GPR101, GNAS, MEN1, CDKN1B, SDHx, and MAX Ectopic expression of GIP (glucose-dependent insulinotropic polypeptide) receptor Overexpression of miR377 and miR136	Pasireotide LAR monotherapy Oral octreotide capsules Pegvisomant Chimeric SST-DA molecules (TBR-065)
**Corticotroph tumors**	Overexpression of miR4501 USP8-mutation ATRX mutation Silent corticotroph tumors	Osilodrostat Pasireotide Mifepristone Ipilimumab + nivolumab Pembrolizumab
**Aggressive pituitary tumors** ** and carcinomas**	Increased expression of pDL2, CE80, and CD86 in pituitary tumor microenvironment Three immune clusters of pituitary adenomas	Temozolomide Anti-VEGF (anti-vascular endothelial growth factor) therapy mTOR inhibitors Tyrosine kinase inhibitors Peptide receptor radionuclide therapy (^111^In-pentreotide, ^177^Lu-DOTA, ^90^Y-DOTA, and ^68^Ga-DOTA) Immunotherapy

## Advances in understanding and managing pituitary adenomas

The introduction of multiomics, including next-generation sequencing genomics, methylomics, transcriptomics, proteomics, and even glycomics, has revealed novel pathways in understanding PA pathogenesis, invasiveness, recurrence, and prognosis.

In 2017, the World Health Organization (WHO) introduced transcription factors for the diagnosis of PAs^4^. Transcriptomic and methylomic analysis has clustered PAs into three groups based on the transcription factor they originate from. The first group, derived from NR5A1, includes clinically non‐functioning PA (CNFPA), gonadotrophinomas, and null cell; the second includes adrenocorticotropic hormone (ACTH) adenomas and silent corticotroph adenomas (SCAs) and is driven by TBX19; the third are the POU1F1‐originating thyroid-stimulating hormone (TSH), prolactin (PRL), and growth hormone (GH) adenomas. Different genes are upregulated in the three groups: in silent ACTH adenomas, gonadotrophinomas, and null cell adenomas (NCAs), genes such as *CACNA2D4* and *EPHB6*; in the clinically functioning ACTH adenomas, the genes *AVPR1B*, *CRHR1*, and *EPHA4*; and in the third cluster, *SLIT1*, *PRLR*, and *SLC16A6*^5^. Moreover, the three groups differ in genes encoding kinases: TBX19-derived adenomas show upregulation of MERTK and STK17B and alterations in VEGFA-VEGFR, EGF-EGFR, and insulin signaling pathways; NR5A1-derived tumors show upregulation of ETNK2 and PIK3C2G and changes in MAPK, ErbB, and RAS signaling; and POU1F1-derived adenomas show upregulation of PIP5K1B and NEK10 and alterations in phosphatidylinositol, insulin, and phospholipase D signaling pathways. By contrast, the expression of genes that encode cyclins and CDK and CDK inhibitors among TBX19, NR5A1, and POU1F1 adenomas is not so different. CDK4 and CDK7 are upregulated in POU1F1 adenomas, but CDK9 and CDK18 are upregulated in NR5A1 adenomas^6^.

Modern methodology has been used to show that the epigenomic landscape differs between the distinct types of PAs^7^. Epigenetic alterations such as DNA methylation, histone modifications, and non-coding RNAs, such as microRNA (miRNA), long non-coding RNAs (lncRNAs), and circular RNAs, are capable of changing the expression of tumor suppressors and oncogenes. These molecules are useful biomarkers and potential therapeutic targets, with the aim of either restoring the expression of tumor suppressor miRNAs or inhibiting the expression of onco-mRNAs^8^.

miRNAs are small protein non-coding RNAs that regulate gene expression post-transcriptionally, and their abnormal expression has been associated with progress of PAs^9^. Divergent lncRNAs have been found in invasive PAs^10^, and different miRNAs are overexpressed in the three PA clusters. The ACTH adenoma group overexpresses miR4501; CNFPA upregulates miR582, miR4774, and *LINC01351*; while the GH, TSH, and PRL adenoma cluster overexpresses miR377 and miR136^5^. Upregulation of spliceosome genes and spliceosome proteins, such as *SRSF1*, *U2AF1*, and *RBM42,* and changes in *CDK18* and *THY1* mRNA have been detected in PAs^11^. *In vitro* experiments of aggressive adenoma tissues unveiled the role of mRNA-146b-5p in suppressing the IRAK4/TRAF6/NF-κB signaling pathway, limiting PA cell progression, and in TMZ-induced chemoresistance *in vitro*^12^.

In 2020, a pangenomic analysis illustrated the DNA hypomethylation in PIT1 lineage and chromosomal instability (except for *GNAS*-mutated somatotrophs) and classified corticotrophs into three classes: the *USP8*-mutated with apparent secretion, the *USP8-*wild-type with increased invasiveness, and the large, silent tumors with gonadotroph transdifferentiation. Unexpectedly, expression of SF-1, which is a transcription factor for gonadotrophs, was also detected in *GNAS-*wild-type somatotrophs^13^.

According to the 2022 update of the WHO classification, acidophil stem cell and mammosomatotroph tumors represent distinct PIT1-lineage pituitary neuroendocrine tumors (PitNETs). Two different PitNETs replace the PIT1-positive plurihormonal tumor category described in the 2017 WHO classification: the mature plurihormonal PIT1-lineage tumor and the immature PIT1-lineage tumor (previously named silent subtype 3 tumor). The term “metastatic PitNET” replaces the term “pituitary carcinoma”^14^.

In 2017, the International Pituitary Pathology Club proposed a change in the term pituitary adenoma to pituitary neuroendocrine tumor PitNET, arguing that pituitary hormone-producing cells are neuroendocrine cells and, in rare cases, their tumors may develop an unusual aggressive behavior similar to that of extrapituitary NETs^15^. The Pituitary Society has disagreed, stating that PAs rarely exhibit a malignant behavior and that labeling them as “NET” generates excessive anxiety and confusion^16^.

In regard to aggressive PAs/carcinomas, TMZ, an oral alkylating chemotherapeutic agent, has been suggested by ESE guidelines as first-line chemotherapy^3^. Meta-analysis data showed that TMZ provokes a 41% radiological overall response and greater biochemical response in functioning adenomas (53%). The 2-year and 4-year survival rates were 79% and 61%, respectively, and survival was prolonged^17^. CAPTEM is a novel combination of capecitabine and TMZ. Capecitabine is an antimetabolite, which enhances the apoptotic effect of TMZ. This novel combination has been used mainly for aggressive corticotroph tumors^18^.

Novel data arise about the role of pituitary tumor microenvironment in the biological behavior of the tumor^19^. This consists of infiltrating the tumor immune cells, cytokines, and chemokines. Differences in immunologic profile between PAs and normal pituitary have been identified. Aggressive PAs show increased expression of pDL2, CE80, and CD86 in comparison with normal human pituitary and higher CD80 and CD86 levels in comparison with non-aggressive tumors. This finding underlines the role of immune checkpoint pathways in PA tumorigenesis and endorses immunotherapy as a novel treatment^20^. Transcriptomic analysis has identified three immune clusters based on tumor infiltration and immune checkpoint molecule expression. CTLA4/CD86 expression was increased in cluster 1, and programmed cell death protein 1/programmed cell death 1 ligand 2 (PD1/PD-L2) expression was enhanced in cluster 2, defining these two groups as “hot” or more responsive to immunotherapy than cluster 3, denoted as “cold”^21^.

Developments in MRI protocols and functional molecular imaging aim to identify PAs and predict their response to treatment^22^. Functional imaging uses radiotracer targeting of either amino acid transport/uptake, such as ^11^C-methionine, or others targeting tumor metabolism, such as ^18^F-FDG, ^13^N-ammonia, and ^18^F-choline. Met-positron emission tomography (Met-PET)/MRI has successfully localized a residual adenoma in 25 out of 26 patients with persistent acromegaly after primary therapy and unclear MRI findings^23^. In a study of 20 patients with ACTH-dependent Cushing’s syndrome, Met-PET/MRI localized adenoma in 7 out of 10 patients with active Cushing’s disease (CD) and in 5 out of 8 with residual or recurrent hypercortisolemia^24^. In a study of 18 patients with intolerant or resistant prolactinomas, MRI and Met-PET/MRI^CR^ findings were in agreement in 14 patients and discrepant in 4, and Met-PET/MRI was false-negative in one patient with a cystic adenoma^25^. Corticotrophin-releasing hormone (CRH) receptor imaging with ^68^Ga-DOTA-CRH represents a novelty in the detection of corticotropinomas. ^68^Ga CRH PET-CT localized a corticotropinoma in 24 cases of CD, including 10 cases with small adenomas smaller than 6 mm (four cases were negative on MRI)^26^. Finally, ^18^F-fallypride may identify dopamine 2/3 receptor expression in prolactinomas and non-functioning PAs^27^.

Lately, peptide receptor radionuclide therapy has entered the therapeutic armamentarium of aggressive PAs/carcinomas that do not respond to conventional treatments. The rationale is based on the expression of somatostatin receptors (SSTRs) on pituitary cells that are detected by functional imaging with octreotide, ^68^Ga-DOTA-TOC or TATE PET and allows radiolabeled somatostatin analogues (SSAs) (^111^In-pentreotide, ^177^Lu-DOTA, ^90^Y-DOTA, and ^68^Ga-DOTA) to be used as targeted therapy. Radiolabeled SSAs enter the cell through binding with the SSTR and release radioagent inside the cell, causing its death. The limited experience so far reports cases with either disease stabilization or partial tumor remission and others that did not respond at all. Two recent reviews have summarized the reported cases and analyzed their results^28,29^.

The development of artificial intelligence and machine learning models, which may process the upcoming amount of medical data, is promising to provide guidance in establishing diagnosis and predict response to treatment^30–32^.

## Non-functioning pituitary adenomas

Non-functioning pituitary tumors lack clinically relevant hormonal excess and represent about 30% of all pituitary tumors^33^. In the 2017 WHO classification, they are divided by immunohistochemistry into NCAs without specific differentiation and clinically silent gonadotrophs, which are hormone-negative PAs and stain for SF-1. NCAs are independently associated with tumor recurrence^34^. Compared with gonadotroph adenomas, NCAs are more invasive at the time of presentation and have a more aggressive clinical course^35^. On the other hand, gonadotroph tumors are heterogeneous. A retrospective analysis of 98 gonadotroph tumors segregated them in follicle-stimulating hormone–luteinizing hormone (FSH-LH), FSH, and LH subtypes and identified male predominance and sex-related differences and association with SSTR (SST2) and estrogen receptor alpha (Erα) expression^36^.

Differences in histone modification and DNA methylation have been observed between NFPAs with and without postsurgical progression^37^, and in comparison with somatotroph adenomas^38^. Three lncRNAs (LOC101927765, RP11-23N2.4, and RP4-533D7.4) have been associated with NFPA recurrence^39^.

Medical treatment of NFPAs is challenging. In an open-label clinical trial that compared cabergoline with non-intervention in patients with residual NFPA after transsphenoidal surgery over 2 years, the progression-free survival rate was significantly higher (23.2 months) in the group of cabergoline in comparison with the control group (20.8 months), and dopamine 2 receptor expression was not associated with cabergoline responsiveness^40^. However, a recent meta-analysis of five studies indicated that cabergoline was more effective in preventing tumor progression than reducing its size^41^.

Treatment with the chimeric compound TBR-760 for 8 weeks in a mouse model of aggressive NFPA resulted in nearly complete inhibition of tumor growth, while treatment with equivalent or higher doses of the individual somatostatin analogue or dopamine, either alone or in combination, had no significant effect^42^. The combination of mTOR inhibitor everolimus with cabergoline *in vitro* has overwhelmed the resistance in the anti-proliferative effects of everolimus on pituitary tumor cell growth through modification of AKT phosphorylation^43^.

## Prolactinοmas

Prolactinomas account for about 40% of all pituitary tumors^2^. Recently, an oncogenic mutation, SF3B1R625H, was identified, leading to a gain-of-function activation of estrogen-related receptor gamma (ESRRG), stronger affinity for Pit-1, and an increase of production of prolactin^44^. Moreover, SF3B1R625H promoted cell migration and invasion through the PI3K/Akt pathway^45^.

Dopamine agonists (DAs) are the first-line treatment for prolactinomas, but a subset presents with medication intolerance or resistance. Interaction between the ER/ERα and PRL/PRLR pathways may contribute to bromocriptine resistance. The combination of bromocriptine with the ERα inhibitor fulvestrant has increased sensitivity to bromocriptine and induced apoptosis in prolactinoma cells^46^.

The expression of SSTRs has been shown in lactotroph adenomas^47^. Octreotide long-acting repeatable (LAR) in addition to cabergoline has shown promising results in patients with resistant prolactinomas^48,49^ and pasireotide, a somatostatin multireceptor ligand, achieved good results in a small number of dopamine-resistant or aggressive prolactinomas^50–52^.

Biguanides exert anti-proliferative and anti-secretory effects in pituitary cells^53^. Metformin reduced prolactin levels and tumor volume in two patients resistant to bromocriptine^54^. On the other hand, in a prospective study of 10 adult patients with resistant prolactinomas (under cabergoline), the addition of metformin failed to show significant results after 6 months, and only two patients showed partial biochemical response^55^.

Alternative therapies with ER modulators, tamoxifen and raloxifen, have shown inconsistent results^56,57^ and their use has not been justified. Aromatase cytochrome P450 enzyme, which aromatizes testosterone to estrogen, is present in normal pituitary tissues and is highly expressed in prolactinoma tissue. It has been related with aggressive behavior and invasiveness of adenomas in both men and post-menopausal women^58,59^. Anastrozole, an aromatase inhibitor, has been added in the therapeutic regimen of four male patients with cabergoline-resistant prolactinomas and prolactin levels decreased (in one case it normalized) while the tumor size decreased by approximately 47% (mean reduction) without serious adverse events^60^.

Dopamine-resistant prolactinomas undergo a β-catenin relocalization in relation to normal pituitaries, and TMZ has downregulated β-catenin and cyclin D1 and markedly reduced prolactin and increased prolactinoma cell apoptosis in mice bearing xenografted prolactinomas, supporting the role of the Wnt/β-catenin pathway^61^. The effect of CAPTEM was assessed in an *ex vivo* culture from two patients with refractory prolactinomas. CAPTEM reduced prolactin levels moderately (9070→4046 ng/mL) in one patient and significantly (17,500→210 ng/mL) in another, while it suppressed the tumor growth in both cases^62^.

Prolactinoma expression of specific ErbB receptors (epidermal growth factor receptors, or EGFRs) is associated with tumor invasion and response to DAs^63^. Lapatinib, a human EGFR2 tyrosine kinase inhibitor, was added on cabergoline therapy of four patients with aggressive prolactinomas. Three out of four patients who had not previously received radiotherapy stabilized their tumor. However, prolactin levels did not normalize, and disease progression occurred^64^.

Combination therapy of ipilimumab plus nivolumab was applied in a case of aggressive prolactinoma, but a further increase of prolactin necessitated a change of therapy to bevacizumab (vascular endothelial growth factor inhibitor), which resulted in stable disease^65^. Pembrolizumab was tried unsuccessfully in a case of metastatic macroprolactinoma, which failed to respond previously to DA therapy and to TMZ and CAPTEM^66^.

The PI3K/Akt/mTOR pathway is an intracellular signaling system which is activated in a significant proportion of prolactinomas. Certain variants of the prolactin receptor, like the Asn492Ile one, are associated with increasing activity of this pathway and cellular proliferation. Everolimus antagonizes these effects *in vitro* and could be a therapeutic option for some aggressive prolactinomas unresponsive to conventional treatments^67^. An off-label trial of everolimus plus cabergoline in a patient with refractory prolactinoma led to significant biochemical response and regression of tumor, which stabilized for 12 months despite a subsequent rise of prolactin levels^68^.

Surgery or radiation are reserved for cases with DA resistance or intolerance, but whether either can be used as first-line treatment has recently been revisited. A meta-analysis demonstrated that 38% of patients who underwent surgery following DA failure achieved remission without the need for further treatment, 62% achieved remission with multimodal treatment, and 16% of cases demonstrated recurrence after an average of 27 ± 9 months^69^. A meta-analysis that compared surgery with medical therapy showed that surgery may achieve long-term remission in the majority of patients (67% vs. 34%), especially in patients with microprolactinoma (87% vs. 36%)^70^. Surgery as a first-line treatment was evaluated by Baussart *et al*. in a well-selected series with non-invasive microprolactinomas and a median follow-up of 18.2 months^71^. Disease-free survival rates at 1 year and 5 years were 90.9% and 81%, respectively, demonstrating that for well-selected microprolactinoma patients, pituitary surgery performed by an expert neurosurgical team is a reasonable option^71^.

Gamma knife radiosurgery (GKRS) normalized prolactin levels in 66.7% of patients in a small cohort (24 patients), 10 patients (41.7%) achieved normal prolactin levels after discontinuation of DAs, six patients (25%) had normal prolactin levels while taking DAs, and all of the patients had tumor control^72^. Similar results arose from a series of 28 patients who had GKRS: normoprolactinemia was achieved in 23 patients (82.1%), normoprolactinemia after discontinuation of DAs was achieved in 13 patients (46.4%), and normoprolactinemia while taking DAs was achieved in 10 patients (35.7%). In all cases, GKRS arrested adenoma growth or decreased adenoma size. In one patient, prolactinoma cystic transformation with expansive behavior, manifested by bilateral hemianopsia, was observed^73^. A meta-analysis of studies on stereotactic radiosurgery of prolactinomas reported tumor control rates of 86% to 100%, new neurological or visual deficit rates of 0% to 5%, endocrine remission rates of 6% to 81%, and new or worsened hypopituitarism in 0% to 62% of patients^74^.

## Somatotroph tumors

Germline mutations (AIP, PRKAR1A, GPR101, GNAS, MEN1, CDKN1B, SDHx, and MAX) and somatic mutations in GNAS are involved in somatotroph tumorigenesis. Moreover, whole-exome sequencing has identified several somatic variants in sporadic GH-secreting PAs without GNAS variants^75^.

AIP participates in the RET-apoptotic pathway in PIT-1-expressing cells. Lack of AIP or pathogenic mutations inhibits this pathway in AIP knockout mice and results in upregulation of somatotroph adenomas^76^. In somatotropinomas with AIP mutations, an overexpression of miR-34a was detected with a subsequent increase of cAMP concentration, cell growth, and reduction of response to octreotide^77^.

Forty percent of somatotroph tumors harbor recurrent activating *GNAS* mutations (the *gsp* oncogene). Multiomics analysis has identified a significant difference between *gsp*-negative and *gsp*-positive tumors in the methylation index. Forty-three percent of *gsp*-negative tumors show *GNAS* imprinting relaxation, which is associated with lower *GNAS*, *SSTR2*, and *AIP* expression, indicating lower sensitivity to somatostatin analogues and potentially aggressive behavior^78^. GNAS mutations are associated with higher preoperative insulin-like growth factor-1 (IGF-1) levels and surgical remission rates and lower nadir GH levels postoperatively^79^.

On the other hand, somatotroph adenomas that show a paradoxical GH increase during the oral glucose tolerance test demonstrate ectopic expression of GIP receptor (GIPR). GIPR-expressing adenomas are negative for activating GNAS mutations, display particular features, and respond better to somatostatin analogues^80–82^. In the future, targeting pituitary GIPR antagonist could be a medical alternative for these adenomas.

Recent epidemiological data show a higher incidence and mortality among females with acromegaly in comparison with males^83,84^. It is now believed that, in biochemically controlled acromegalic patients, mortality is similar to the general population and the risk of comorbidities is lower, although structural deformities are unlikely to reverse. Recent series have shown reduced severity of cardiovascular disease in patients with acromegaly and reallocate cancer as the main cause of death; this is possibly due to better control of the disease and longer life expectancy^84^.

Predictors of response to medical treatment have been identified. A meta-analysis reported that clinical predictors of response to first-generation somatostatin analogues for treatment-naïve patients are female gender, older age, and lower baseline IGF-1 as well as GH less than 1.2 μg/L and IGF-1 less than 110% of the upper limit of normal (ULN) at 12 weeks after initiation of first-generation SSAs. This response relates to biochemical control at 12 months^85^. Hypointense adenomas on T2 MRI signals respond better with greater IGF-1 reduction and tumor shrinkage after lanreotide^86^. Extension of dosing intervals with lanreotide autogel 120 mg (6- or 8-weekly dosing) has been effective in patients previously biochemically controlled with octreotide LAR 10 or 20  mg/4 weeks^87^.

For cases in which lanreotide or octreotide LAR fail to control the disease, switching to pasireotide LAR monotherapy is an option, but worsening of glycemia should be taken into consideration, especially for those with impaired fasting blood glucose at baseline^88^. Recently, the first oral SSTR ligand was approved by the US Food and Drug Administration for acromegaly. Oral octreotide capsules (OOCs) are considered an effective treatment for patients with acromegaly who have previously responded well to injectable somatostatin analogues^89,90^. In the OPTIMAL trial, a phase 3 randomized controlled trial, the primary endpoint of IGF-1 of not more than 1×ULN was achieved in 58% of the OOC cohort versus 19% in the placebo cohort^89^. As recommended in the Pituitary Society Update for the management of acromegaly, dose initiation of OOC is 40 mg/day given twice a day as a capsule of 20 mg at fasting state or 2 hours postprandial. Uptitration is followed by 20 mg every 2 to 4 weeks according to IGF-1 levels and clinical evaluation^84^.

According to the ACROSTUDY, pegvisomant, the GH receptor antagonist, can biochemically control almost 73% of patients, and only 3.2% of patients have abnormal transaminases, and 6.8% show a tumor size increase on MRI^91^. Pegvisomant improves glucose metabolism independently of IGF-1 control^92^.

For patients uncontrolled after maximum dose of SSAs therapy, combination of low-dose octreotide LAR or lanreotide plus weekly pegvisomant is a cost-effective treatment that may achieve biochemical control in almost 95%^93^. In the PAPE study, the combination of pasireotide plus pegvisomant achieved high control rates even above 70%, but the frequency of diabetes doubled^94^.

Compared with individual SST or DA analogs (alone or combined), chimeric SST-DA compounds (dopastatins) are more potent in inhibiting GH secretion. Compared with currently available therapies, TBR-065 has significantly improved efficacy in suppressing GH secretion^95^.

## Corticotroph tumors

Corticotroph cells give rise to ACTH-secreting adenomas resulting in CD, clinically silent ACTH adenomas (SCAs), Crooke cell adenomas (CCAs), and ACTH-producing carcinomas (CAs). SCAs lack clinical and biochemical features of hypercortisolemia but show a high recurrence rate and do not respond well to common treatment^96^. A study that compared SCAs with silent gonadotroph adenomas showed that the two groups had comparable size and recurrence/progression rates but SCAs were more invasive and had cystic changes^97^. Different expression of miRNAs has been identified in clinically functioning adenomas and SCAs. High expression of hsa-miR-124-3p in adenomas causing CD may be involved in the regulation of feedback by corticosteroids at the glucocorticoid (GC) receptor level^98^.

A genomic study of 27 corticotroph tumors classified them in a USP8-mutated group, genome-stable with little somatic copy number variation (sCNV) and a USP8-wild type group, genome-disrupted, with TP53 mutations and extensive somatic copy number variation (sCNV). *USP8*-mutated tumors exhibited nonaggressive behavior as four out of five tumors were microadenomas and only one *USP8*-mutated tumor developed metastasis, which responded well to therapy^99^. Whole-exome sequencing of one patient with an ACTH-producing CA, one CCA, one corticotrophinoma occurring in a CD patient who developed Nelson syndrome after adrenalectomy, three SCAs, and four ACTH-secreting PAs causing CD showed that the neoplasm with the highest number of genomic abnormalities was the ACTH-CA, followed by the CCA and the CD tissues. The ACTH-CA and the four clinically functioning ACTH adenomas showed more copy-number variation (CNV) gains and single-nucleotide variations (SNVs) than the non-functioning tumors and shared the amplification of 10q11.22. Nevertheless, all of the tumors shared some genomic abnormalities, which is an indication of a common biological spectrum^100^. Mutations in the alpha thalassemia/mental retardation syndrome X-linked (ATRX) gene, which regulates chromatin remodeling and telomere maintenance, have been detected in aggressive corticotroph tumors^101^.

As far as it concerns clinical issues, the recent consensus on diagnosis and management of CD has updated recommendations and presented relevant algorithms^102^. It highlighted that no single diagnostic test is adequate for diagnosis and that diagnostic testing should be individualized on the basis of clinical criteria. Testing includes the assessment of (i) circadian rhythm by late night salivary cortisol (LNSC) or midnight serum cortisol, (ii) cortisol feedback by the dexamethasone suppression test, and (iii) 24-hour bioavailable cortisol by urinary free cortisol (UFC).

It was recommended that for differential diagnosis from ectopic ACTH Cushing syndrome, patients with pituitary lesions of less than 6 mm should proceed to inferior petrosal sinus sample (IPSS), for tumors 6 to 9 mm most experts suggested that IPSS is performed, but for lesions of at least 10 mm this is not necessary. Comorbidities (hypercoagulability, cardiovascular disease, bone disease, and infections) should be managed adequately even before the specific treatment of hypercortisolemia. Transsphenoidal surgery is the first-line treatment and may achieve high remission rates (defined as postoperative cortisol of less than 2 μg/dl) when performed in specialized Pituitary Tumor Centers of Excellence (PTCOE) by an experienced pituitary neurosurgeon. Lifelong monitoring for recurrence of CD is mandatory as recurrence rate may reach 35%. Assessment of recurrence follows the same tests as in diagnosis. LNSC is the most sensitive and should be repeated on an annual basis after the recovery of hypothalamic–pituitary–adrenal (HPA) axis. Postoperative dynamic desmopressin testing with ΔCort of less than 7.4 μg/dl has a sensitivity of 97% to detect remission but its value applies only in patients with a documented preoperative positive test^103^. It is based on the fact that tumorous corticotrophs aberrantly express V2 receptors but that normal corticotrophs do not. Persistence of the response postoperatively indicates residual tumor.

Medical therapy for CD targets adrenal steroidogenesis (ketoconazole, metyrapone, mitotane, etomidate, and osilodrostat), somatostatin and dopamine receptors in the pituitary, and GC receptors. Usually, adrenal steroidogenesis inhibitors are introduced first and in severe cases combinations can be used.

Osilodrostat is a new, potent oral inhibitor of 11β-hydroxylase (CYP11B1) approved in Europe for the treatment of endogenous Cushing’s syndrome and in the US for CD. In LINC 3, a phase III study, patients with confirmed persistent or recurrent CD complete response (mean 24-hour UFC concentration of not more than ULN) with osilodrostat at week 34 was achieved in 86% versus 29% (placebo). Hypocortisolism occurred in 51% of patients, and adverse events related to adrenal hormone precursors occurred in 42% of patients^104^. In the LINC 4 study, significantly more patients on osilodrostat (77%) achieved mean UFC (mUFC) of not more than ULN than placebo (8%) patients at week 12, and response was maintained at week 36, when 81% of all patients achieved mUFC of not more than ULN. Notably, patients with diabetes improved their glycemia status during osilodrostat treatment. Moreover, clinical features were improved.

In cases of severe hypercortisolemia where rapid normalization of cortisol is wanted, etomidate, an anesthetic agent, can be used in the intensive care unit.

Pasireotide is the first medical therapy officially approved for adult patients with CD. In a phase 3 study, long-acting pasireotide normalized mUFC concentration in about 40% of patients with CD at month 7 and had a safety profile similar to that of twice-daily subcutaneous pasireotide^105^.

The GC receptor blocker mifepristone is effective in controlling some effects of hypercortisolism, regardless of the cause. The Study of the Efficacy and Safety of Mifepristone in the Treatment of Endogenous Cushing Syndrome (SEISMIC) phase 3 study showed improvement in glycaemia in about 60% of patients and a decrease of diastolic blood pressure in 38%. As cortisol concentrations remain high during treatment with mifepristone, monitoring is based on clinical signs^106^.

In rare cases, corticotroph tumors may become aggressive. A patient with an aggressive, sparsely granulated corticotroph adenoma and malignant transformation did not respond to TMZ despite loss of expression of MLH1 and PMS2 in the tumor cells. Next-generation sequencing using the MSK-IMPACT platform identified somatic mutations in MLH1 Y548lfs*9 and TP53 R337C. Immunotherapy using ipilimumab/nivolumab was initiated, and no residual tumor was seen in MRI 34 months postoperatively^107^. Another patient with a treatment-refractory aggressive ACTH-secreting pituitary carcinoma responded to ipilimumab and nivolumab^108^. Majd *et al*. described the effects of pembrolizumab in two patients with functioning corticotroph pituitary carcinomas (refractory to surgery, radiotherapy, and chemotherapy) who had partial radiographic (60% and 32% per Immune-Related Response Evaluation Criteria in Solid Tumors, respectively) and hormonal responses^66^. They had previously received TMZ. Patient 1’s response continued 42 months after initiation of pembrolizumab and his tumor tissue, which was obtained after treatment with TMZ, demonstrated a hypermutator phenotype with *MSH2* and *MSH6* gene mutations. A third patient with a silent corticotroph tumor (patient 3) unresponsive to TMZ stabilized disease for 4 months after immunotherapy^66^.

## Conclusions

In this review, we summarized the recent major advances in the field of understanding and managing PAs. There is currently a need to obtain knowledge of the molecular biology of PAs as this will open new perspectives in understanding their pathogenesis, predicting their recurrence, and managing these tumors effectively. PAs are the second most common intracranial neoplasm and are associated with significant morbidities and mortality, especially in the rare case of aggressive tumors. Multiomic technology in the field of molecular biology, radiology, and pathology creates an innovative, promising field for their diagnostic and therapeutic management. Understanding the different activated molecular pathways in comparison with normal pituitary is an important step toward the development of targeted therapies and their effective management.
